# Study on the Wood Characteristics of the Chinese Ancient Ship Luoyang I

**DOI:** 10.3390/ma16031145

**Published:** 2023-01-29

**Authors:** Xinyou Liu, Wanrong Ma, Xinwei Tu, Houyi Huang, Anca Maria Varodi

**Affiliations:** 1 Co-Innovation Center of Efficient Processing and Utilization of Forest Resources, Nanjing Forestry University, Nanjing 210037, China; 2 College of Furnishing and Industrial Design, Nanjing Forestry University, Str. Longpan No.159, Nanjing 210037, China; 3 Faculty of Furniture Design and Wood Engineering, Transilvania University of Brașov, 500036 Brasov, Romania; 4 Advanced Analysis and Testing Center, Nanjing Forestry University, Str. Longpan No.159, Nanjing 210037, China

**Keywords:** microscopy, waterlogged archaeological wood, SEM, XRD, FTIR, nanoindentation

## Abstract

Luoyang No.1 is a Qing Dynasty (1644–1902) inland river ancient wooden shipwreck discovered in September 2013. It adds significantly to the study of Grand Canal transport history and Luoyang’s economic history. The wood characteristics of Luoyang No.1 were investigated in this study using chemical compositions, X-ray diffraction (XRD), Fourier transform infrared spectroscopy (FTIR), nano-indentation (NI), and scanning electron microscopy (SEM). The results showed that the holocellulose content was only 32.84–37.69%, indicating that the cellulose and hemicellulose had been seriously degraded. Based on the XRD pattern, the degree of crystallinity of cellulose in wood ranged from 19.82 to 22.83%. The nano-indentation demonstrated that compared with the undegraded contemporary wood, the elastic modulus and hardness of the ancient ship wood decreased by 45.5% and 32.1%, respectively. Furthermore, the FTIR spectra revealed that the biological deterioration of ancient wood was indicated by a decrease in the peaks related to cellulose and hemicellulose, but the change in lignin was insignificant. The results could provide knowledge for appropriate dewatering, strengthening, restoration strategies and regulation of the museum environment.

## 1. Introduction

**Figure 1:**
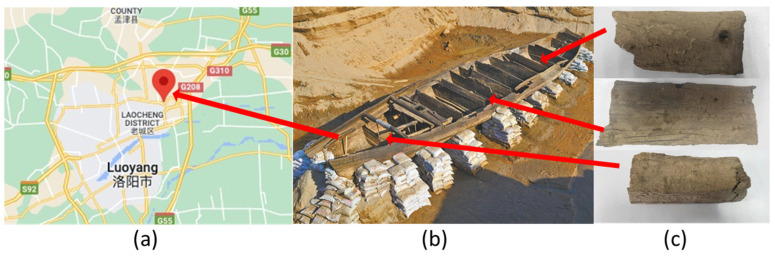
Basic information of Luoyang No.1 ancient Ship: (**a**) Excavation location; (**b**) ancient ship; (**c**) Wood samples extracted from keels of ancient ships.

Luoyang I is a shipwreck discovered in September 2013 in Shouyangshan Village (E112°38′30.7″, N34°42′33.8″) (Figure 1a), Yanshi Town, Luoyang City, and Henan Province. The sunken ship was discovered in the old Grand Canal channel of the Sui and Tang Dynasties (581–907), which is also named old Yangqu channel of Luoyang in modern time [1]. The study of the Grand Canal’s transport history and Luoyang’s economic history is of significant relevance. The entirety of the ship was uncovered after about seven months of excavation and cleaning. The ancient ship is now being transferred to the Luoyang Museum, awaiting further restoration and conservation. The sunken ship was discovered waterlogged at the bottom of the channel, buried in the sand at a depth between 5.7 m and 6.2 m. It has 13 compartments and is 20.14 m long and 3.48 m wide, with a maximum of 1.42 m remaining (Figure 1b). Carbon-14 analysis of excavated porcelain, iron products, wood, and soil confirmed that the shipwreck occurred during the early Qing Dynasty [2]. The wreckage wood has deteriorated due to microbial infestation in the underground environment, so the study of the wood’s characteristics will guide the next step in restoration and conservation.

The assessment of the preservation state of the waterlogged archaeological wood contributes to a better understanding of the decay mechanism and is the basis for designing a conservation strategy [3,4,5,6]. The state of preservation of waterlogged archaeological wood is affected by many factors, such as wood species, decaying agent [7], storage conditions [8,9], and pre-burial condition [10,11]. When using a single analysis, each analysis has limitations. Over the past few decades, multidisciplinary diagnostic analyses, including micromorphology, physical and chemical characteristics, have been developed to accurately estimate waterlogged archaeological wood preservation status. At present, multi-analytical techniques have been widely recognized, such as scanning electron microscopy (SEM) [12,13], X-ray diffraction (XRD) [14], infrared spectroscopy (FTIR) [15], X-ray photoelectron spectroscopy (XPS) [16], thermogravimetric analysis (TGA) [17], and nano-indentation (NI) [18,19]. Micromorphological analysis can explore detailed structural decay characteristics of wood cell walls and provide descriptive results on degradation [20]. When correlated with qualitative parameters obtained through physical and chemical analysis, a complete approach was developed to gain insight into waterlogged archaeological wood.

The degradation related chemical and physical changes in waterlogged archaeological wood cell walls causes the fragility of cell wall, and degraded cell walls tend to collapse due to capillary forces and the high surface tension of evaporating water [21,22]. Therefore, only dewatering with special dehydration methods can maintain dimensional stability during the drying process of water-immersed wood, ethanol replacement, PEG replacement, freeze-vacuum drying [23,24], supercritical carbon dioxide [25,26,27,28]. Polyethylene glycol (PEG), trehalose, polyoctadecanol, epoxy resin, and other materials with good stability are commonly used in ancient wood consolidation [29,30,31,32,33,34].

This research aimed to examine the wood characteristics of the ancient wreck “Luoyang I”. Determination of chemical composition, X-ray diffraction (XRD), Fourier transform infrared spectroscopy (FTIR), nano-indentation (NI), and scanning electron microscopy (SEM) were used to investigate the degradation state of archaeological wood. This research will be helpful for the further restoration and conservation of the ancient shipwreck from inland river.

## 2. Materials and Methods

### 2.1. Materials

Three pieces of wood samples were collected from various parts (Figure 1c) of the keel of the Luoyang No.1 ancient ship, obtained from the Luoyang Institute of Cultural Relics and Archaeology and identified as cork oak (*Quercus variabilis*). Before the specimen was extracted, the water-saturated wood from the excavated site was treated as follows: A 4% borine-based WP-1 aqueous solution was sprayed with a PEG compound solution (7% urea + 21% dimethyl urea + 10% PEG 4000). The concentration of the PEG compound solution (up to 40%) gradually increased until the wood reached PEG saturation. Although these archaeological woods were treated with PEG at the excavation site, they need to be further treated after being transferred to the museum to restore the sunken ship. In this study, the initial moisture content was 76.84 ± 7.69% by the GB/T1931–2009 [35] method for the determination of the moisture content of the wood. These samples were then dried at 40 °C with an RH of 30% for two weeks.

### 2.2. Determination of Chemical Compositions

In this study, undegraded contemporary wood from the same wood spices cork oak (*Quercus variabilis*) with 70 years of tree age and 3 years of harvested, obtained from Jeson wood company (Huzhou, China) was used as a reference to evaluate the deterioration of the ancient ship wood. The chemical compositions of surface ancient wood (SW), core ancient wood (CW), and undegraded contemporary wood (UC) were determined. Based on GB/T2677.6–94 Fibrous Raw Material—Determination of Solvent Extractives, the content of solvent extractives [36] was measured. Using the standard GB/T2677.10–94 Fibrous raw material determination of holocellulose [37], we measured holocellulose content.

### 2.3. X-ray Diffraction

After drying and cleaning the surface until the wood grain was visible, wood samples were extracted from the surface layer of approximately 2 mm, the core layer of the ancient wood, and undegraded contemporary wood as a control. They were ground to 80-mesh powders and pressed into three sample sheets (three wood samples for each case) at room temperature. Wood samples were investigated using in situ XRD with an X’Pert Pro multipurpose diffractometer (PANalytical, Almelo, The Netherlands) and Rigaku Smart Lab 9 kW XRD system (Shimadzu Corporation, Kyoto, Japan). In addition, θ-2θ scanning was used to measure the role of scattering intensity and scattering angle. The angle range is 5–40°, with the scanning speed being 2°/min. The spectrum provided was the average of three measurements for the undegraded contemporary and ancient wood. Using the Segal method, the height of the (002) peak (I_002_, 2θ = 22.8°) and the minimum value between the (002) and (101) peaks (I_AM_, 2θ = 18°) were used to determine the crystallinity of cellulose using the following equation:

CR_x_ = (I_002_ − I_AM_)/I_002_ × 100% (1)

where CR_x_ (%) denotes the degree of crystallinity of cellulose, I_002_ denotes both crystalline and amorphous materials, and IAM indicates an amorphous material.

### 2.4. Chemical Composition Analysis

Wood samples from the surface layer (SW), core layer (CW), and undegraded contemporary wood (UC) were ground and passed through an 80-mesh sieve, and pressed into KBr pellets for FTIR investigation. The FTIR test equipment was an IR spectrometer (Tensor 27, Bruker, Ettlingen, Germany) with a spectral resolution of 4 cm^−1^ within the range of 4000 cm^−1^ and 400 cm^−1^ for a total of 32 scans. FTIR spectra of each wood sample were recorded six times, further processed for baseline correction and smoothing. An average spectrum of the six individually recorded ones was calculated. This average spectrum was further normalized (max–min normalization). These normalized average spectra of SW, CW and UC were further compared to highlight chemical changes due to the degradation.

### 2.5. Quasi-Static Nano-Indentation Test

The moisture content of the ancient wood and undegraded contemporary wood could be adjusted to 12 ± 2% before nano-indentation testing. Five samples not embedded in any resin of the CW and UC were cut into 7 mm × 5 mm × 5 mm prisms, with the top and bottom made parallel using the procedure outlined by Meng et al. [38]. An ultramicrotome with a diamond knife was used to ensure a smooth test surface. This experiment used an in-situ nano-indentation systems (Hysitron TI980, Bruker, Ettlingen, Germany) with the diamond Berkovich tip. Moreover, this experiment was carried out in load function mode using a three-segment load ramp; the load application was 5 s, the hold time was 5 s, and the unloading time was 5 s. In addition, the peak load was 400 N for all indents in the test, resulting in a maximum penetration depth of 150–200 nm in the wood cell wall. Under room temperature and relative humidity, five indentations on the cell wall of latewood were made for the ancient and modern wood samples.

Based on the nano-indentation theory, hardness (*H*) and reduced elastic modulus (*Er*) were computed from the load-displacement data using the equations below, as depicted by Oliver and Pharr [39]:

(2) $$H=\frac{P_{max}}{A}$$

where *P_max_* is the peak load detected at a maximum depth in an indentation cycle, and *A* is the projected contact area between the indenter and the sample.

The sample’s reduced elastic modulus (*E_r_*) can then be computed as follows:

(3) $$E_{r}=\frac{\sqrt{\pi }}{2}\frac{S}{\sqrt{A}}\;\;$$

where *S* (stiffness) indicates the slope of the line of the unloading curve in the load-displacement plot, and *A* is the projected contact area. *S* was determined using a linear approximation of the high-load portion of the unloading curve (ranging from 90% to 70% of the load).

## 3. Results and Discussions

### 3.1. Determination of the Main Component Content

The content of alcohol-benzene extraction from ancient wood (5.67–8.69%) was much higher than that of undegraded contemporary wood (2.67%). This is mainly due to anaerobic bacteria that corrupt the wood when starved underwater. Similarly, the concentration of 1% NaOH extract of ancient wood was higher than that of undegraded contemporary wood, and the value of the surface layer was higher than that of the core layer because the content of urea on the surface was higher than that in the core layer after pretreatment. The low holocellulose content was only 32.84–37.69%, indicating that cellulose and hemicelluloses can be significantly degraded. Therefore, the relative lignin content of wood samples reaches 53.24–56.48%, which is much higher than that of undegraded contemporary wood (Table 1).

### 3.2. X-ray Diffraction

**Figure 2:**
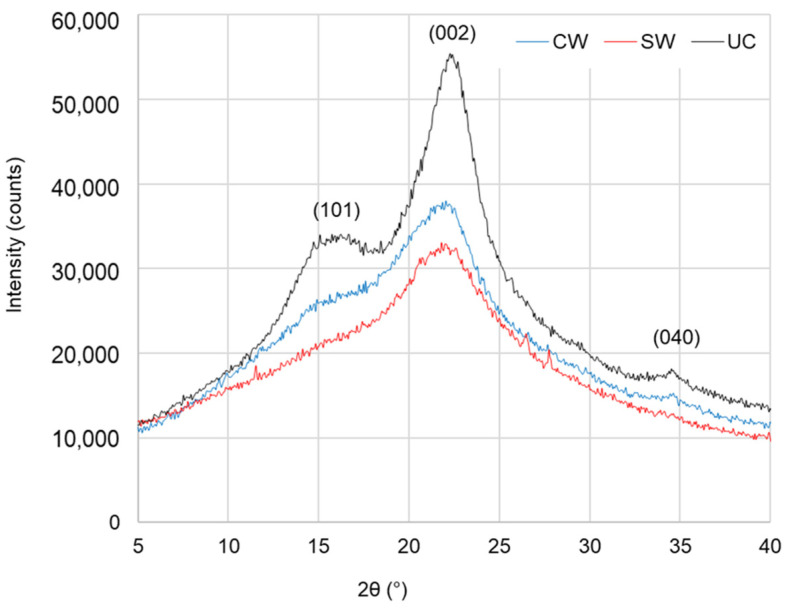
X-ray diffraction pattern of ancient shipwreck wood from Luoyang No.1 and undegraded contemporary wood.

Cellulose is one of the three main components of wood. Crystallinity reflects the cellulose microfilament structure and is typically measured using an X-ray diffractometer [40]. Figure 2 presents X-ray diffraction patterns of ancient wood (surface layer and core layer) from Luoyang No.1 and undegraded contemporary wood. Only the diffraction pattern of an undegraded contemporary wood sample has three peaks at 2θ of 18°, 22.5°, and 35°, conforming to (101), (002), and (040) crystal planes separately. The other two patterns of archaeological wood only have the maximum diffraction peak of (002) around 2θ = 22.8° and a very sight peak near 2θ = 18° due to the severe degradation. Compared with the intensities of these diffraction patterns, undegraded contemporary wood > ancient wood core layer > ancient wood surface layer caused by different cellulose content [41]. Following Equation (1), the calculated crystallinity degree of cellulose for the undegraded contemporary wood sample reaches 40.28%, but those of the surface layer and core layer of ancient wood are 22.83 and 19.82%. The decreased crystallinity in ancient wood is due to cellulose decrystallisation [42]. In anoxic or nearly anoxic waterlogged conditions, biodeterioration of the wood is mainly bacterial, while more oxygenated environments additionally facilitate decay caused by soft-rot fungi [43]. According to the excavation conditions as oxygenated environment, it can be judged that the loss of wood cellulose of the ancient ship may be caused by the invasion of soft-rot fungi.

### 3.3. Chemical Structure Analysis Using FTIR Spectroscopy

**Figure 3:**
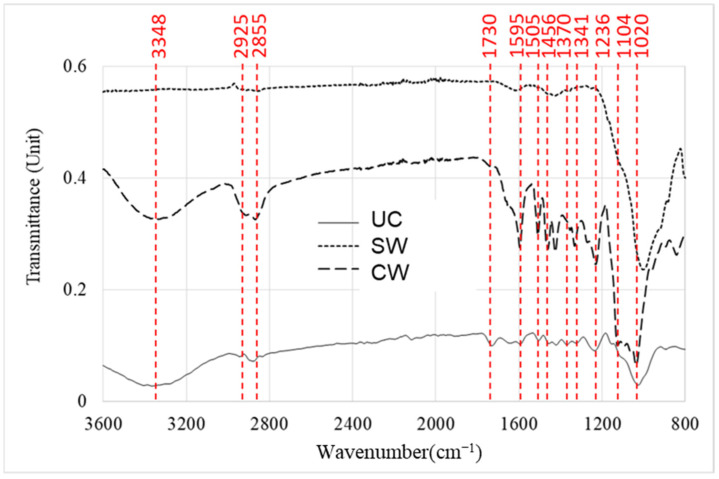
FTIR spectra of ancient shipwreck wood from Huaguang Jiao I.

Compared with the spectra of undegraded contemporary wood and the core layer of ancient, the spectrum of the ancient wood surface has a different pattern (Figure 3), resulting from the PEG-treatment, which has an intense peak around 1000 cm^−1^ due to C-O stretching vibration in PEG 4000 [28,43]. Compared with the undegraded contemporary wood, the spectrum of CW has a strong band 1020 cm^–1^ due to C-O stretching vibration slightly shifted, which can also illustrate the effect of PEG 4000 pretreatment [44]. The most evident difference between spectra is the complete disappearance in SW samples of unconjugated carbonyl groups at around 1730 cm^−1^. This peak was associated with chromophores resulting from cellulose and hemicellulose degradation [15]. When compared UC and CW, it can be observed also the changes in the absorption intensity of 1730 cm^−1^ peak, this is mainly because the wood from a 2 mm layer on the surface was degraded more severely than in the core layer were the peak was almost disappeared (in SW). The decrease of peaks at around 1157 cm^−1^ assigned to asymmetric C-O-C stretching, associated with cellulose and hemicelluloses, indicating an apparent decrease in holocellulose content [45]. In contrast, changes in the absorption intensity of the peak at 1595 cm^−1^ conforming to the C=C stretching of the aromatic ring (lignin) [46] can be observed. However, the peaks at 1505 cm^−1^ did not change significantly, indicating that lignin slightly degraded during the deterioration process, which is in agreement with previous literature [47].

### 3.4. Nano-Indentation Test

**Figure 4:**
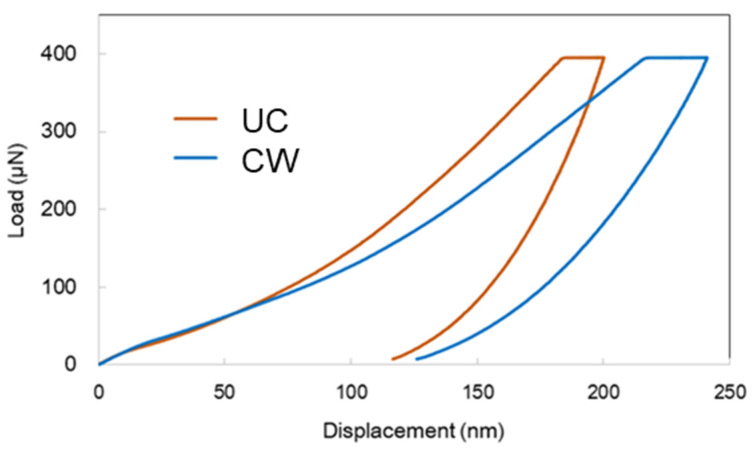
Typical NI load-displacement curves of ancient wood and undegraded contemporary wood.

Nano-indentation tests the change in wood mechanical properties at the nano-scale, calculates the elastic modulus and hardness through load and displacement changes [38]. The typical nano-indentation load-displacement curves, of ancient and undegraded wood presented in Figure 4 suggest that the shapes of curves related to the mechanical strengths were different. Under the same large load, the displacement of the ancient wood was significantly greater than that of the undegraded contemporary wood. According to Table 2, the mean elastic modulus of the ancient wood is 5.83 GPa, with a 45.5% reduction from the healthy modern wood of 9.87 GPa. On the other hand, the mean hardness value of ancient wood was 0.36 GPa, with 32.1% smaller that the undegraded contemporary wood of 0.53 GPa. Therefore, after natural deterioration, the mechanical strength of ancient wood was significantly reduced by 32.1–45.5%. The decrease in mechanical strength of ancient wood is due to the loss of cellulose and hemicellulose in the cell wall [17,19], which leads to the fragile cell wall, which will seriously increase the difficulty of dehydration and consolidation of ancient wood [18]. This fact leads to a great challenge for restoration and conservation.

## 4. Conclusions

The wood of the ancient shipwreck Luoyang No.1, dating from the Qing Dynasty (1644–1902), was compared to an undegraded contemporary wood of the same species. The ship wood showed mainly degradation of cellulose and hemicelluloses, concentrating the more stable lignin. A change in the intensity of the C=O structure indicated the degradation of xylan hemicelluloses. The cellulose crystal phase also went under intense degradation. The surface of the wood had a more severe degradation of hemicelluloses, while the cellulose crystallinity degraded more in the core wood. The loss of quantity and quality of the wood’s main chemical components caused a sharp reduction in its mechanical strength. The findings obtained in these studies could provide valuable information for the restoration and conservation of this ancient ship.

## Figures and Tables

**Table 1:** Chemical composition of Luoyang No.1 ancient shipwreck wood.

Wood Samples	Alcohol-Benzene Extract (%)	1%NaOH Extract (%)	Acid Accumulator Insoluble Lignin (%)	Holocellulose (%)
SW	8.69	18.57	56.48	32.84
CW	5.67	14.56	53.24	37.69
UC	2.67	7.84	31.69	79.23

**Table 2:** Nanoindentation test results of ancient wood and healthy modern wood.

Wood Samples	Elastic Modulus (GPa)	Hardness (GPa)
Ancient wood	5.83 (0.36)	0.36 (0.02)
Undegraded contemporary wood	9.87 (0.24)	0.53 (0.03) ^1^

^1^ The average values of the 25 measurements and standard deviations are in brackets.

## Data Availability

Not applicable.
